# Hospitalization with hypoglycemia in patients without diabetes mellitus: A retrospective study using a national inpatient database in Japan, 2008–2012

**DOI:** 10.1097/MD.0000000000007271

**Published:** 2017-06-23

**Authors:** Akahito Sako, Hideo Yasunaga, Hiroki Matsui, Kiyohide Fushimi, Hidetaka Hamasaki, Hisayuki Katsuyama, Tetsuro Tsujimoto, Atsushi Goto, Hidekatsu Yanai

**Affiliations:** a Department of Internal Medicine, Kohnodai Hospital, National Center for Global Health and Medicine, Chiba; b Department of Clinical Epidemiology and Health Economics, School of Public Health, Graduate School of Medicine, The University of Tokyo; c Department of Health Informatics and Policy, Tokyo Medical and Dental University Graduate School of Medicine; d Department of Diabetes, Endocrinology, and Metabolism, Center Hospital, National Center for Global Health and Medicine; e Metabolic Epidemiology Section, Epidemiology and Prevention Group, Center for Public Health Sciences, National Cancer Center, Tokyo, Japan.

**Keywords:** body mass index, hospitalization, hypoglycemia, mortality, nondiabetes, prevalence

## Abstract

We aimed to examine prevalence, patient characteristics, etiology, and clinical outcomes of hospitalized patients who had hypoglycemia without a diagnosis of diabetes mellitus, using a Japanese nationwide database.

This was a retrospective observational study using a national database of acute-care inpatients in Japan. Nondiabetic patients aged ≥15 years who were hospitalized for hypoglycemia were eligible. We estimated the annual numbers of hospitalized cases in Japan. We also investigated the patient characteristics, and risk factors of in-hospital mortality.

We identified 8684 eligible patients out of 22.7 million discharge records between July 2008 and March 2013. The average age was 70.0 years and the average body mass index (BMI) was 19.9 kg/m^2^. Most frequently recorded underlying diseases were malignancies, cerebrovascular diseases, pneumonia, renal failure, and heart failure. The estimated annual numbers of hospitalizations because of hypoglycemia in nondiabetic patients were 5000 to 7000. In-hospital mortality was 14.9%, and predictive factors for poor survival included older age, community hospital, low BMI, coma at admission, urgent admission, renal failure, heart failure, pneumonia, sepsis, chronic liver diseases, and malignancies.

Patients without diabetes mellitus but with hypoglycemia had multiple comorbidities and high in-hospital mortality. Clinicians should carefully investigate the etiology of hypoglycemia in nondiabetic patients, and treat the underlying diseases.

## 1 Introduction

Hypoglycemia is a common clinical event in diabetic patients who use hypoglycemic agents.^[1]^ Severe hypoglycemia in diabetic patients leads to increased cardiovascular events and mortality.^[2–4]^ Recent clinical trials showed that hypoglycemia in critically ill patients without diabetes mellitus was associated with poor clinical outcomes.^[5–7]^ These findings address the harm of severe hypoglycemia and challenge the intensive glycemic control strategy for the management of both diabetic patients^[8]^ and critically ill patients.^[9]^ Although hypoglycemia is uncommon in patients without diabetes, a wide variety of etiologies can cause hypoglycemia, including sepsis, liver diseases, malnutrition, alcohol-related diseases, malignancies, postgastrectomy syndrome, and endocrine disorders.^[1,10,11]^ To our knowledge, nationwide studies on severe hypoglycemia in nondiabetic patients that investigated the prevalence, patient characteristics, and clinical courses are lacking. Most previous studies were based on case series of hypoglycemia in single centers or large-scale multicenter studies confined to critically ill patients.

We conducted a large-scale retrospective observational study using an administrative and clinical inpatient database to determine the prevalence, patient characteristics, and clinical outcomes of hospitalization with hypoglycemia in nondiabetic patients in Japanese acute-care hospitals.

## 2 Materials and methods

### 2.1 Diagnosis Procedure Combination (DPC) database

The DPC database includes discharge abstract and administrative claims data.^[12,13]^ Participation in the database is mandatory for all 82 academic hospitals in Japan, while it is not mandatory for community hospitals. Data collection periods were 6 months per year (between July and December) until 2010. Since 2011, data have been collected all year round. As of 2012, the number of participating hospitals was 1098, and the total number of beds was 388,000. This figure represented 43% of all the beds in acute-care hospitals in Japan. The numbers of hospital admissions recorded in the database were 2.82 million in 2008, 2.78 million in 2009, 3.30 million in 2010, 6.96 million in 2011, and 6.85 million in 2012. The number in 2012 represented approximately 50% of all admissions from Japanese acute-care hospitals. The present study was a secondary analysis of administrative claims data. Informed consent was not required due to the anonymous nature of the data. The Institutional Review Board at The University of Tokyo approved the study.

### 2.2 Patient selection and variables

The DPC data contain a maximum of 12 diagnoses. They were recorded according to International Classification of Diseases 10th Revision (ICD-10) codes and Japanese text data. The recorded diagnoses consist of 4 main diagnoses, 4 comorbidities at admission, and 4 complications during hospitalization. We retrospectively extracted the records for patients who had hypoglycemia-related ICD-10 codes (Table 1) in the 4 main diagnoses from the fiscal years 2008 to 2012. We confirmed the diagnoses using the text data as needed. We excluded patients aged under 15 years and patients with diabetes. We identified patients with diabetes by any ICD-10 code for diabetes (E10–E14) in the diagnoses, or prescription of oral hypoglycemic agents or glucagon-like peptide-1 analogs. We did not exclude patients who used insulin, because insulin could be administered for nondiabetic patients. Finally, only nondiabetic patients aged ≥15 years hospitalized with hypoglycemia were eligible for the present study.

**Table 1:**
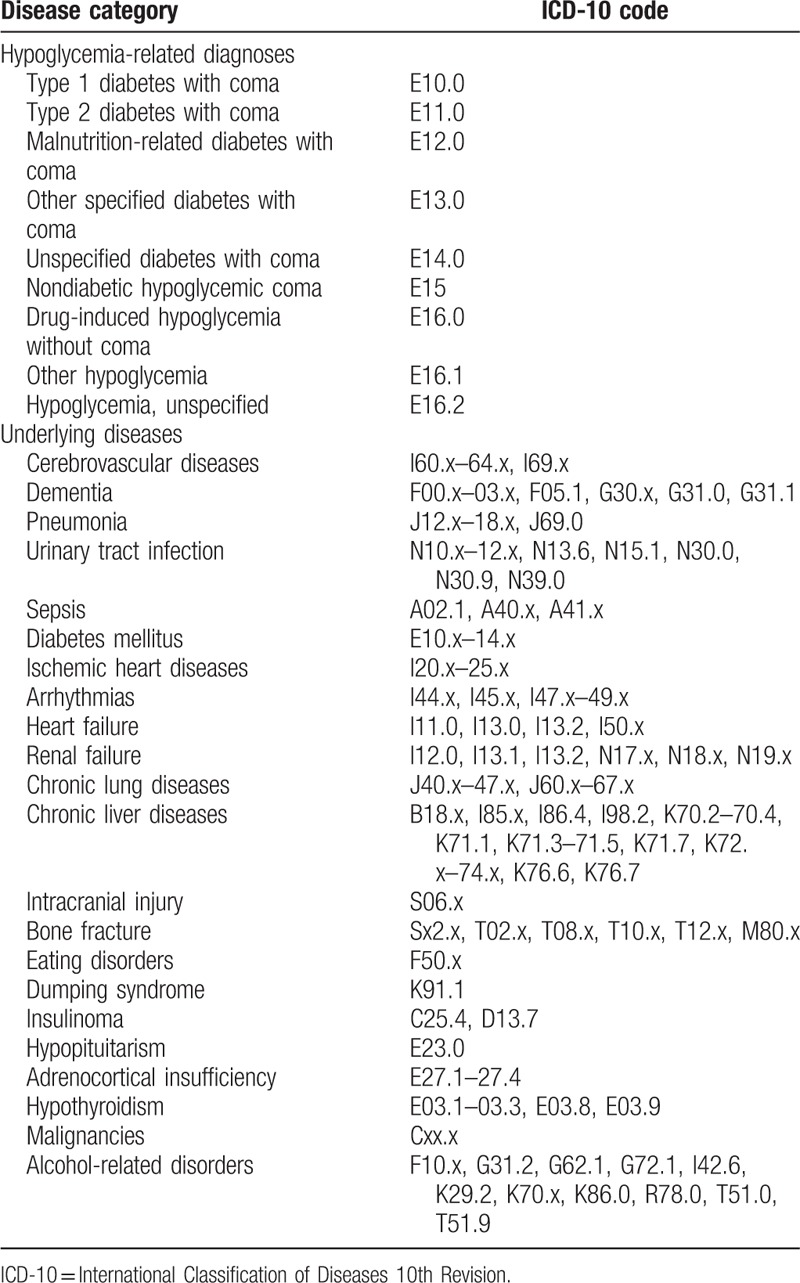
ICD-10 codes for hypoglycemia and underlying diseases.

We collected the following data: age at admission; sex; body weight and height (available since 2010); discharge status; ambulance use; unique hospital identifier; hospital type (academic or community); number of hospital beds capacity; underlying diseases (Table 1); procedures and surgeries; medications prescribed during hospitalization; medical care costs; and dates of hospital admission and discharge. Body mass index (BMI) was calculated according to the following standard equation: BMI  =  body weight in kilograms/height in meters squared. BMI was divided into 5 groups^[14]^: underweight, BMI < 18.50 kg/m^2^; low-normal weight, 18.50 to 22.99 kg/m^2^; high-normal weight, 23.00 to 24.99 kg/m^2^; overweight, 25.00 to 29.99 kg/m^2^; obese, ≥30.00 kg/m^2^. BMI data were not available for inpatients from 2008 to 2009. Consciousness levels were evaluated at admission and discharge based on the Japan Coma Scale (JCS), and categorized into 4 groups^[15]^: grade 0, alert; grade 1, drowsy, but awake without any stimuli; grade 2, somnolence, but arousable with stimulation; grade 3, coma. We examined the burden of comorbidities based on the Charlson Comorbidity Index (CCI) using the protocol of Quan et al.^[16,17]^

### 2.3 Estimated prevalence of hospitalization with hypoglycemia in nondiabetic patients

The number of hospitalized hypoglycemia in Japan in each fiscal year (Yi) was estimated using the following equation:
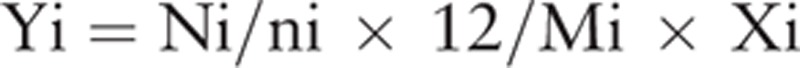


where Ni is the number of beds capacity in all acute-care Japanese hospitals,^[18]^ ni is the number of beds capacity in the DPC participating hospitals, Mi is the number of months for submission of DPC data, and Xi is the observed annual number of nondiabetic hypoglycemia cases in the DPC hospitals. Because the DPC hospitals were skewed toward larger hospitals, we stratified hospitals by bed-volume for the adjustment. The 95% confidence interval (CI) for each year was computed using the Wald CI for the population proportion.

### 2.4 Statistical analysis

We performed multivariable logistic regression analysis to determine the factors associated with mortality during hospitalization and the odds ratios and 95% CIs. In the multivariable regression model, the independent variables included age, sex, and variables which were clinically relevant and significantly associated with in-hospital death in univariate analyses. To adjust for clustering effect by hospital, statistical model was fitted with a generalized estimating equation.^[19]^ Variance inflation factors for independent variables were calculated to assess multicollinearity between the independent variables. A variance inflation factor >10 was considered to represent multicollinearity. Patients with missing BMI data were categorized into the group with “missing data.” In the multivariable logistic regression analysis, we used data for all patients, including the group with missing BMI data. We did not use complete case analysis (which excludes patients with missing data) due to the risk of introducing bias. Values of *P* < .05 were considered statistically significant. All statistical analyses were performed by IBM SPSS Statistical package Version 20 (IBM Corp., Armonk, NY).

## 3 Results

### 3.1 Patient demographic data

Among 22.7 million discharge records for the 45 months from July 2008 to March 2013, we identified 81,433 patients who had any ICD-10 code for hypoglycemia in their 12 diagnoses. Of these, 8684 patients were eligible for the present study based on our inclusion criteria. The patients’ characteristics are shown in Table 2. The average age was 70.0 years (SD: 17.4) and the average CCI was 2.5 (SD: 2.5). The average height, body weight, and BMI were 157.0 cm (SD: 10.0), 49.2 kg (SD: 13.0), and 19.9 kg/m^2^ (SD: 4.5), respectively. Approximately 40% of patients had missing BMI data.

**Table 2:**
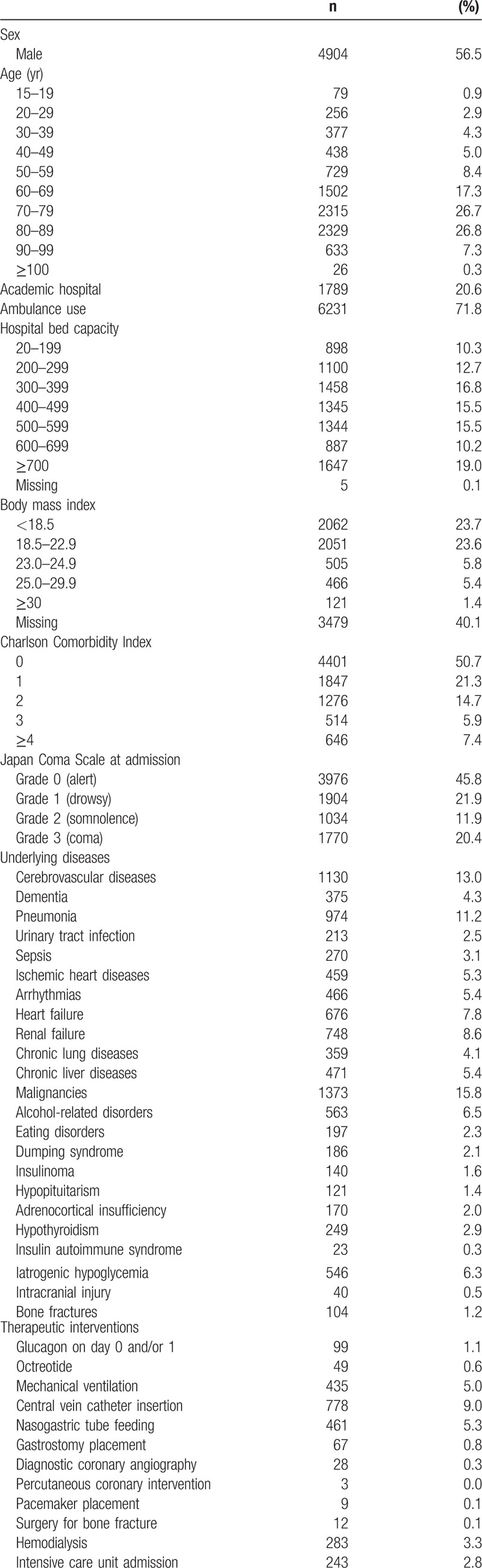
Patient characteristics (n  =  8684).

### 3.2 Prevalence of hospitalization with hypoglycemia in nondiabetic patients

The estimated annual cases of hospitalizations with hypoglycemia in Japan were 5000 to 7000 (Table 3).

**Table 3:**
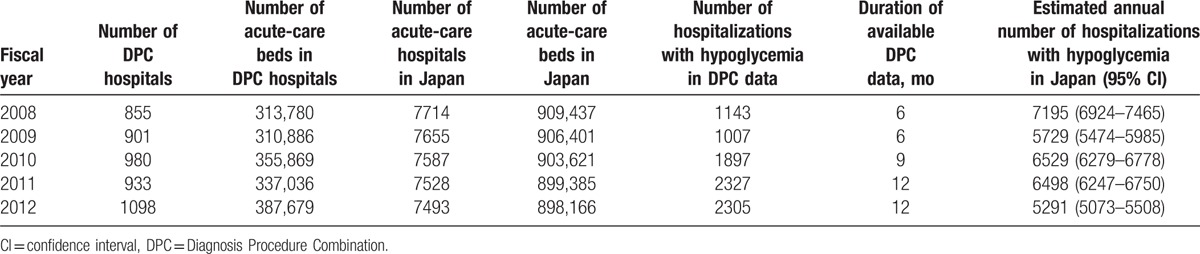
Estimated annual numbers of hospitalizations with hypoglycemia in nondiabetic patients in Japan.

### 3.3 Clinical outcomes and risk factors for in-hospital mortality

The median length of hospitalization was 6 days (interquartile range: 13 days). The median medical care cost was 228,000 JPY (interquartile range: 411,000 JPY; 1 USD  =  80–100 JPY during the study period). In terms of functional status at discharge without death, enteral tube feeding was present in 2.4% of patients, parenteral nutrition by central venous catheter was present in 1%, and JCS was Grade 0 in 79.0%, Grade 1 in 9.7%, Grade 2 in 1.2%, Grade 3 on 0.7%, and missing in 9.4%.

The overall in-hospital mortality was 14.9%, the proportion of patients discharged to home was 71.1%, and the proportion of patients discharged to places other than home was 13.9%.

The results of the multivariable logistic regression analysis for in-hospital mortality are shown in Table 4. Variance inflation factors were <10 for all the independent variables. The factors associated with poor survival were older age, community hospital, low BMI, coma at admission, urgent admission, renal failure, heart failure, pneumonia, sepsis, chronic liver diseases, and malignancies. Dumping syndrome, iatrogenic hypoglycemia, hypopituitarism, cerebrovascular diseases, and alcohol-related disorders were associated with lower in-hospital mortality.

**Table 4:**
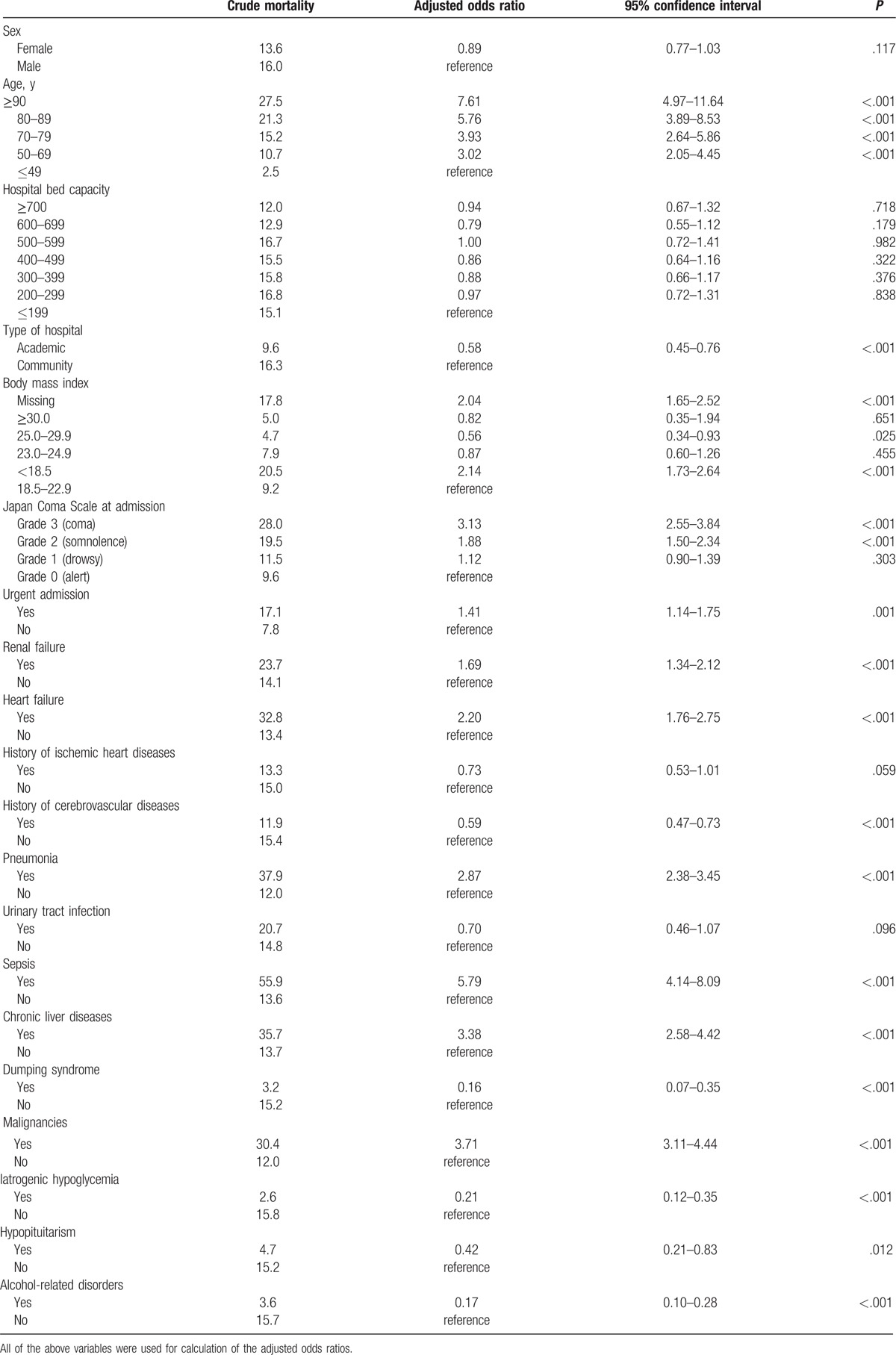
Logistic regression analysis for in-hospital mortality.

## 4 Discussion

The present nationwide retrospective observational study involving 8684 hospitalized hypoglycemia without diabetes examined both epidemiological and clinical data in acute-care hospitals in Japan. The average age of the patients was 70.0 years, and 78% were aged >60 years. The in-hospital death was 14.9%, and predictive factors for poor survival were older age, nonacademic hospital, low BMI, coma at admission, urgent admission, renal failure, heart failure, pneumonia, sepsis, chronic liver diseases, and malignancies.

There have been several nationwide studies on hospitalization for diabetic hypoglycemia.^[20–22]^ However, to the best of our knowledge, there are only 2 previous single-center studies on hospitalization for hypoglycemia without diabetes^[10,11]^: one was undertaken in a tertiary care hospital in Japan and the other in a university hospital in UK, but the sample sizes were relatively small and consequently the findings may lack generalizability. Most of the clinical guidelines for hypoglycemia are limited to patients with diabetes, except for the guideline of the Endocrine Society in the United States published in 2009.^[1]^ Therefore, comprehensive clinical and epidemiological data have been lacking. The present study is the first nationwide research on hospitalization with hypoglycemia in nondiabetic patients.

In our previous study using the DPC database, we found that the estimated annual numbers of hospitalizations for diabetic hypoglycemia in Japan were 16,000 to 22,000.^[20]^ Combined with the present study, nondiabetic hypoglycemia accounted for 20% to 30% of hospitalizations with hypoglycemia, and these figures were not negligible. In a previous study involving 59,602 consecutive ambulance visits to the emergency room of a tertiary-care hospital in Japan, 530 cases had severe hypoglycemia including 163 nondiabetic cases (31%).^[10]^ The proportions of nondiabetic patients hospitalized with hypoglycemia were similar between the previous study and the present study. A previous study in the United Kingdom found that the frequency of nondiabetic hypoglycemia outside the intensive care units in university hospitals was 50 per 10,000 admissions using 3 data sources, namely blood glucose <3.3 mmol/L, medication with high-strength glucose solution or glucagon, and diagnostic codes for hypoglycemia.^[11]^ The frequency with a cut-off of 2.2 mmol/L was 8 per 10,000 admissions. In our study, we found 8684 nondiabetic hypoglycemia cases among 22.7 million discharge records, giving a frequency of 3.8 per 10,000 admissions. This lower frequency in the present study was partly because we did not include patients with hypoglycemia during hospitalization.

A wide range of diseases can cause hypoglycemia in nondiabetic patients.^[1]^ Although our study could not identify the true causes of the hypoglycemia, malignancies, cerebrovascular diseases, infection, major organ failure, and alcohol-related disorders were the most frequently recorded underlying diseases, and these are known to cause hypoglycemia. The common comorbidities linked with hypoglycemia were sepsis, kidney diseases, and alcohol dependence followed by pneumonia, liver diseases, cancer, and self-harm with hypoglycemic agents in the UK study.^[11]^ In a Japanese tertiary-care hospital, malnutrition was the leading cause of nondiabetic hypoglycemia followed by alcohol, infection, and postgastrectomy.^[10]^ Little is known about the differences in patient characteristics between nondiabetic hypoglycemia and diabetic hypoglycemia. We previously described the characteristics of patients hospitalized for diabetic hypoglycemia.^[20]^ The mean age of the patients was 73.4 years, and about 90% were aged >60 years. The mean BMI was 22.3 kg/m^2^, with 17% of patients considered underweight according to their BMI. Compared with diabetic hypoglycemia, the patients with nondiabetic hypoglycemia were younger and had lower BMI.

In previous single-center studies, the in-hospital mortality for hypoglycemic hospitalizations was 1.6% to 4.9% in diabetic patients,^[21,22]^ 7.1% to 11% in mixed populations,^[23,24]^ and as high as 15.9% to 33.8% in nondiabetic patients.^[11,25]^ An Italian nationwide study showed that the in-hospital mortality due to hypoglycemic coma was 2.1%.^[26]^ To our knowledge, only one previous study directly compared the mortality rates of severe hypoglycemia between nondiabetic and diabetic patients.^[10]^ The study showed that the nondiabetic patients had significantly higher mortality within 90 days after severe hypoglycemia than the diabetic patients (22.1% vs 1.6%). Age, preexisting advanced liver diseases and cancer, coexistence of sepsis, and blood glucose <40 mg/dL were predictors of death in nondiabetic patients. Comparing the present and previous studies,^[20]^ nondiabetic hypoglycemia had higher mortality than diabetic hypoglycemia in a large-scale nationwide cohort (14.9% vs 3.8%). Although we could not determine whether hypoglycemia was a direct cause of death or a clinical indicator of severity, hypoglycemia is a known independent risk factor for death in critically ill patients.^[5–7]^ The present study showed that lower BMI was more common in nondiabetic hypoglycemia than in diabetic hypoglycemia, and also a predictive factor for in-hospital mortality. The frequently recorded comorbidities such as malnutrition, chronic organ failure, and malignancies might account for the lower BMI. Several studies showed a U-shaped association between BMI and mortality.^[27,28]^ Although previous studies revealed that diabetic patients with BMI <18.5 kg/m^2^ had significantly higher risk of death than diabetic patients with normal and overweight,^[29,30]^ the present study revealed that lower BMI with nondiabetic hypoglycemia was significantly associated with higher in-hospital mortality for the first time.

In this study, patients with missing BMI data were categorized into a “missing data” group. In the multivariable logistic regression analysis, we used data for all patients, including the group with missing BMI data. We did not use complete case analysis (which excludes patients with missing data) as the proportion of patients with missing BMI data was high, and to have excluded them may have resulted in biased results.

The present study has several limitations. First, the DPC database does not contain some clinical information, including vital signs and laboratory data such as plasma glucose and HbA_1c_. Because we selected the eligible patients based on the main diagnoses, we may have overlooked hypoglycemic cases with severe concurrent diseases at admission such as severe sepsis, hypothyroidism, and renal failure even if patients also had hypoglycemia. Therefore, our study may have underestimated the annual numbers of hospitalizations with hypoglycemia. Second, the DPC database is limited to admission to acute-care hospitals, and participating hospitals are skewed toward large bed-capacity academic hospitals. To adjust for this bias, we stratified hospitals by bed-capacity categories. Third, the DPC database only contains in-hospital information and is not linked with other databases such as outpatient records and vital statistics. As a result, we could not investigate prescriptions before admission and survival after discharge, and our results are limited to short-term clinical course and in-hospital mortality. In spite of these limitations, our nationwide study has determined the annual prevalence, patients’ demographics, and in-hospital mortality of nondiabetic hypoglycemia hospitalizations in an Asian population, and confirmed the results from previous single-center studies.

In conclusion, the present study showed that nondiabetic hypoglycemia accounted for a significant proportion of hospitalizations with hypoglycemia. The in-hospital mortality was as high as 14.9% and higher than that of diabetic hypoglycemia. The disease burden and clinical importance were not ignorable. Clinicians should carefully examine the etiology of hypoglycemia in patients without diabetes mellitus, and treat the underlying causes.
